# Etude de l'anémie chez les enfants séropositifs au VIH naïfs au traitement antirétroviral à Lubumbashi, République Démocratique du Congo

**DOI:** 10.11604/pamj.2014.17.46.2046

**Published:** 2014-01-22

**Authors:** Costa Kazadi Mwadianvita, Eric Kasamba Ilunga, Jackson Djouma, Cecile Watu Wembonyama, Florence Mujing A Mutomb, Michel Balaka Ekwalanga, Joe Kabongo, Henri Mundongo, Kalombo Mupoya, Stanis Wembonyama, Prosper Kalenga Mwenze, Albert Mwembo-Tambwe A Nkoy

**Affiliations:** 1 Département de Sciences Biomédicales, Faculté de Médecine de l'Université de Lubumbashi, Lubumbashi, République Démocratique du Congo; 2 Centre d'Excellence de Prise en Charge des Personnes vivant avec le VIH/SIDA, Lubumbashi, République Démocratique du Congo; 3 Département de Santé Publique, Faculté de Médecine de l'Université de Lubumbashi, Lubumbashi, République Démocratique du Congo; 4 Département de Pédiatrie, Faculté de Médecine de l'Université de Lubumbashi, Lubumbashi, République Démocratique du Congo; 5 Département de Gynécologie Obstétrique, Faculté de Médecine de l'Université de Lubumbashi, Lubumbashi, République Démocratique du Congo

**Keywords:** Anémie, enfants séropositifs au VIH/SIDA, Lubumbashi, RD Congo, anemia, children with HIV / AIDS, Lubumbashi, Congo Democratic Republic

## Abstract

**Introduction:**

Beaucoup d'enfants infectés par le VIH arrivent à la consultation dans un état d'anémie. Notre objectif était d’évaluer la prévalence et le typage de l'anémie chez ces enfants.

**Méthodes:**

C'est une étude transversale réalisée dans 3 centres de prise en charge des Personnes Vivant avec le VIH à Lubumbashi de Mai 2010 à Mai 2011. La population d’étude était de 152 enfants, âgés de 6 à 180 mois, naïfs au traitement antirétroviral. Les statistiques descriptives usuelles ont été utilisées.

**Résultats:**

La prévalence globale de l'anémie (définie comme l'hémoglobine < 11g/dl) était de 69,1% (n=105) et 11,4% avaient une anémie sévère (Hg < 7,0 g/dl). Parmi eux, 16% ont été transfusés au moins 1 fois. L'anémie sévère était positivement associée au stade clinique de la maladie (p=0,02). L'anémie microcytaire était majoritaire dans les deux tranches d’âge. Elle était plus hypochrome chez les enfants en âge préscolaire soit 9,5% et plus normochrome en âge scolaire soit 15,2%. L'anémie normocytaire était plus normochrome dans les deux tranches d’âge soit 12,4% en âge préscolaire et 6,7% en âge scolaire. L'anémie macrocytaire était rare.

**Conclusion:**

Environ sept enfants sur dix, âgés de moins de 15 ans infectés par le VIH naïfs au traitement antirétroviral dans notre milieu sont anémiques. L'anémie est corrélée à la sévérité de la maladie. Il est important d'associer une prise en charge nutritionnelle et corriger l'anémie avant une trithérapie antirétrovirale.

## Introduction

L'anémie demeure une des complications hématologiques la plus fréquente du VIH [1]. Sa prévalence est en nette progression soit 73-100% des cas d'anémie chez les enfants infectés par le VIH dans le monde en 2008 [2]. En Afrique, Calis et al. [3] ont rapporté en 2008 une prévalence de 53% au Malawi.

En République Démocratique du Congo (RDC), une enquête menée en 2007 par EDS-RDC (Demographic and Health Survey) a montré que 71% d'enfants âgés de 6-59 mois présentaient une anémie [4]. A Lubumbashi (RDC), Ngwej et al. ont rapporté une prévalence de 75% en 2007 [5].

Bon nombre de facteurs responsables de l'anémie chez les enfants VIH+ ont été déterminés et certaines stratégies d′intervention ont été proposées. Pourtant, force est de constater que l'anémie figure toujours parmi les principales causes de morbidité et de mortalité au monde ; en Afrique mais aussi en RDC où le nombre d'enfants vivant avec le VIH/SIDA n'est pas négligeable (33000 à 86000 en 2009) [6]. La question qui se pose est celle de savoir quelle est actuellement l'ampleur de l'anémie chez ces enfants âgés de moins de 15 ans à Lubumbashi.

Ce travail avait pour objectif d’évaluer la prévalence et le typage de l'anémie chez les enfants séropositifs du VIH/SIDA à Lubumbashi.

## Méthodes

**Cadre et type de l’étude:** Cette étude a été menée à Lubumbashi, en RD Congo, dans trois(3) centres de prise en charge des Personnes Vivant avec le VIH(PVV) notamment le Centre d'Excellence(CE) de l'hôpital Sendwe, le Centre Amo-Congo de la Kenya et l'hôpital général de Référence de la Kenya. Ces trois formations médicales assurent les consultations et la prise en charge des PVV sans exclusivité d’âge et de sexe. Il s′agit d′une étude descriptive transversale qui s′est déroulée sur une période de 12 mois (Mai 2010 à Mai 2011).

**Population d’étude:** Elle est constituée de 152 enfants séropositifs au VIH, âgés de 6 à 180 mois repartis en âge scolaire (≥72 mois) et en âge préscolaire (< 72 mois), tous naïfs au traitement antirétroviral et dont le bilan biologique a été fait systématiquement. Les enfants ont été diagnostiqués et classés selon des catégories cliniques et immunologiques en fonction de critères de l′OMS [7]. La prise en charge en ambulatoire des enfants comprenait la surveillance clinique et biologique avant la mise sous traitement, et tous les enfants sont sous prophylaxie au cotrimoxazole.

**Recueil des données et analyses statistiques:** Les variables suivantes ont été prélevés : l’âge, le sexe, le stade clinique de la maladie, les infections opportunistes (I.O), la notion d'une transfusion antérieure.

**Paramètres biologiques:** Les analyses suivantes ont été effectuées sur le sang total à l'aide d'un automate Micro 60 du fabricant HORIBA ABX Diagnostic (2005) au laboratoire des cliniques Universitaires de Lubumbashi : Hémoglobine (Hg), Hématocrite (Hct), Globules Rouges, Réticulocytes, Volume Globulaire Moyen(VGM), Concentration Corpusculaire Moyenne en Hb (CCMH).

**Classification de l'anémie:** L'anémie a été définie selon les critères de l'OMS par un taux d'hémoglobine inférieur à 11 g/dl. Elle est considérée comme sévère à un taux d'hémoglobine inférieur à 7,0 g/dl. Elle est modérée si ce taux se situe entre 7,0 g/dl et 9,9 g/dl. Et l'anémie est considérée comme légère si ce taux se situe entre 10 g/dl et 11g/ [8]. Une anémie microcytaire, normocytaire et macrocytaire ont été définies chez les enfants en âge préscolaire lorsque le volume globulaire moyen(VGM) est respectivement de < 73, 73-80, > 80 femto litres (fl.) et l'hypochromie lorsque la concentration corpusculaire moyenne en hémoglobine (CCMH) est <32% chez les enfants en âge préscolaire. La microcytose, la normocytose et la macrocytose ont été définies lorsque le VGM est respectivement de <80, 80-98,>98 chez les enfants en âge scolaire [9].

**Analyse des données:** Les données ont été saisies et analysées à l'aide du logiciel SPSS 11.0, Epi Info version 3.5.1 et Excel 2007. Pour la comparaison de proportion, le Chi carré corrigé de Fisher était utilisé avec un seuil de signification de p < 0,05.

## Résultats

### Caractéristiques des enfants infectés par le VIH

La population étudiée était constituée de 152 enfants dont 52,6% (n=80) du sexe féminin et 47,2%(n=72) du sexe masculin. Le sex-ratio est de 0,9. L’âge moyen était de 68,32±3,6 mois dont 46,1% (n=70) en âge scolaire et 53,9 % (n=82) en âge préscolaire. En fonction du stade clinique, 24,3% d'enfants étaient au stade avancé de la maladie (stade III et IV selon l'OMS 2006).

### Prévalence et caractéristiques de l'anémie chez les sujets infectés par le VIH

La prévalence globale de l'anémie était de 69,1% (n=105). La moyenne de l'hémoglobine était de 9,90g/dl (avec le minimum de 3,70g/dl et le maximum était de 16,20g/dl) et 16% des enfants anémiques ont été transfusés au moins une fois. La moyenne de l'hématocrite était de 29,03% (11,20-46,50). La moyenne des globules rouges était de 3.804.605,26/mm3 (1.550.000- 6.050.000). En fonction de la tranche d’âge, 23,38%(n=48) des enfants en âge scolaire étaient anémiques contre 32,09%(n=57) en âge préscolaire.

Douze sujets (11,4%) présentaient une anémie sévère et 54,3% une anémie modérée (Tableau 1). S'agissant de la sévérité de l'anémie par rapport au stade clinique, elle était associée à l’évolution de l'infection à VIH (X^2^ =14,9 et p= 0,02) (Tableau 2).

**Tableau 1:** Répartition des patients selon la sévérité de l'anémie

Sévérité de l'anémie	Effectif (n)	Pourcentage (%)
Sévère	12	11,4
Modérée	57	54,3
Légère	36	34,3

L'analyse de ce tableau montre que 11,4% présentaient une anémie sévère et 88,6% une anémie modérée et légère

**Tableau 2:** La sévérité de l'anémie selon le stade clinique

Sévérité de l'anémie	Stade Clinique			
	1 n (%)	2 n (%)	3 n (%)	4n (%)
Anémie sévère	2 (4,7)	2 (6,5)	6 (21,4)	2 (66,7)
Anémie modérée	25 (58,1)	17 (54,8)	14 (50,0)	1 (33,3)
Anémie légère	16 (37,2)	12 (38,7)	8 (28,6)	0

L'analyse de ce tableau montre que la sévérité de l'anémie étaient associée à l’évolution de l'infection à VIH (X^2^ =14,9 et p= 0,02).

### Typage de l'anémie chez les enfants infectés par le VIH

**Figure 1:**
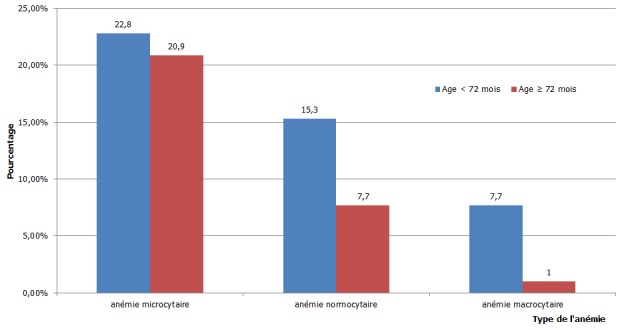
Répartition de l'anémie selon le volume globulaire moyen par tranche d’âge

La Figure 1 montre que tous les enfants présentaient plus une anémie microcytaire dans les deux tranches d’âge (âge préscolaire : 22,8% (n= 24) et âge scolaire : 20,9% (n=22) et moins une anémie normocytaire. L'anémie macrocytaire était rare.

L'analyse de la morphologie des globules rouges a montré que tous les enfants quelle que soit la tranche d’âge, présentaient plus une anémie microcytaire. Elle était plus normochrome (28,5%) qu'hypochrome (15,2%) dans les deux tranches d’âge. L'anémie normocytaire était plus normochrome (19,1%) qu'hypochrome (3,9%) dans les deux tranches d’âge. Les enfants en âge préscolaire présentaient plus une anémie macrocytaire quelle soit hypochrome (4,8%), normochrome (2,9%) que les enfants en âge scolaire (1%) (Tableau 3).

**Table 3:** Répartition des enfants par tranche d’âge selon le type d'anémie

Type d'anémie	Enfants âgés de 〈72 mois n (%)	Enfants âgés de 〉72 mois n (%)
Anémie microcytaire hypochrome	10 (9,5)	6 (5,7)
Anémie microcytaire normochrome	14 (13,3)	16 (15,2)
Anémie normocytaire hypochrome	3 (2,9)	1 (1,0)
Anémie normocytaire normochrome	13 (12,4)	7 (6,7)
Anémie macrocytaire hypochrome	5 (4,8)	0
Anémie macrocytaire normochrome	3 (2,9)	1 (1,0)

L'analyse de la morphologie des globules rouges a montré que tous les enfants quelle que soit la tranche d’âge, présentaient plus une anémie microcytaire normochrome.

## Discussion

Dans notre étude, 69,1% d'enfants VIH+ suivis ont été anémiques. Plusieurs études dont celle Farhan M et al. [10] et Anita Shet et al. [11] ont signalé tous en 2009 une fréquence similaire à la nôtre soit 62% et 66% en Inde alors que des chiffres encore plus élevés ont été signalé soit 70-100% une prévalence mondiale [2], 90% en Ouganda [12], 77,9% au Nigeria [13] et 73% en Afrique du Sud [14]. Nos résultats corroborent ces observations.

Nos résultats rejoignent les observations faites par Ngwej et al. (2007) qui avaient signalé une prévalence de 75% à Lubumbashi [5]. Cinq ans après, elle demeure la complication hématologique la plus fréquente chez les enfants infectés par le VIH dans notre milieu.

Dans notre étude, nous avons trouvé 11,4% des cas d'anémie sévère et 54,3% des cas d'anémie modérée alors que Anita Shet et al. ont signalé en 2009 une fréquence légèrement faible soit 8% des cas d'anémie sévère en Inde [11]. Cette sévérité de l′anémie était positivement associée au stade avancé de la maladie (p < 002). De même, Anita Shet et al. rapportèrent une constatation similaire chez les enfants infectés par le VIH [11]. Dans le contexte de l'infection à VIH, l'inflammation chronique, la carence en micronutriments et les infections opportunistes aggravent l'anémie chez ces enfants.

L'anémie microcytaire a été objectivée chez les enfants infectés par le VIH quelle que soit la tranche d’âge avec une légère prédominance à l’âge préscolaire soit 22,8% contre 20,9% à l’âge scolaire, probablement due à la vulnérabilité de ces enfants à développer l'anémie en raison d'une grande fréquence d′infections gastro-intestinales dans cette tranche d’âge. Cette anémie microcytaire était plus normochrome (35,7%) qu'hypochrome (11,2%) alors que Farhan M. (2009) a signalé une anémie plus normocytaire normochrome (33,8%) suivie par une anémie microcytaire hypochrome (24,3%) en Inde [10]. Les anémies microcytaires sont les formes les plus fréquentes dans notre milieu. Par contre, les anémies macrocytaires étaient rares.

La présence des IO chez les enfants peut encore aggraver l′anémie. Swaminathan S et al. (2008) ont constaté en Inde que les enfants séropositifs au VIH atteints de tuberculose pulmonaire étaient trois fois plus susceptibles de développer l′anémie et que la co-infection TBC-VIH est un puissant facteur de risque pour l′anémie, notamment l′anémie sévère [15]. La faible proportion des cas de tuberculose dans notre étude ne nous a pas permis d’évaluer cette co-infection.

L’étiologie spécifique dont la recherche des parasitoses intestinales et du paludisme n'a pas été abordée dans cette étude comparativement à d'autres études [16]. Cette étude rapporte des données épidémiologiques importantes sur l'anémie chez les enfants infectés par le VIH en République Démocratique du Congo et met en évidence, la nécessité d'une approche prospective afin de comprendre l'ampleur et l’étiologie du problème de manière à planifier des interventions appropriées.

## Conclusion

L'anémie doit être considérée à Lubumbashi comme une préoccupation majeure, pour les enfants séropositifs du VIH/SIDA. Le stade avancé de l'infection à VIH y est associé dans une grande proportion. La surveillance des paramètres hématologiques chez les enfants infectés par le VIH/SIDA s'avère indispensable afin de détecter au plus tôt les anomalies dans le but de trouver l’étiologie et le traitement approprié.

## References

[1] Mocroft A, Kirk O, Barton SE, Dietrich M, Proenca R, Colebunders R (1999). Anaemia is an independent predictive marker for clinical prognosis in HIV-infected patients from across Europe. EuroSIDA Study Group..

[2] Calis JC, van Hensbroek MB, de Haan RJ, Moons P, Brabin BJ, Bates I (2008). HIV-associated anemia in children: a systematic review from a global perspective.

[3] Calis Job, Hellen P, Rotteveel, Rotteveel P, Antoinette C van der Kuyl (2008). L'anémie sévère n'est pas associé à-gène env : caractéristiques 1 VIH chez les enfants du Malawi. Maladies infectieuses BMC.

[4] Demographic and Health Survey (2007). Rapport d'enquête en République Démocratique du Congo.

[5] Ngwej T (2007). Profils clinique et biologique de l'infection à VIH chez l'enfant :cas des cliniques universitaires.

[6] ONUSIDA (2010). Rapport sur l’épidémie mondiale du SIDA.

[7] WHO (2006). Case definitions of HIV for surveillance and revised clinical staging and immunological classification of HIV-related disease in adults and children.

[8] WHO (2002). Iron Deficiency Anaemia: Assessment, Prevention and Control. A Guide for Programme Managers.

[9] Irwin JJ, Kirchner JT (2001). Anemia in Children. Am Fam Physician.

[10] Farhan M Hematological manifestations in children with HIV/AIDS.

[11] Anita Shet, Saurabh Mehta, Nirmala Rajagopalan, Samuel NM (2009). L'anémie et des troubles de croissance chez les enfants infectés par le VIH en Inde: une analyse rétrospective. BMC Pediatrics.

[12] Totin D, Ndugwa C, Mmiro F, Perry RT, Jackson JB, Semba RD (2002). L'anémie ferriprive est très fréquente chez les virus de l'immunodéficience humaine, infectés ou non, les nourrissons en Ouganda. Journal Nutr..

[13] Adetifa IM, Temiye EO, Akinsulie AO, Ezeaka VC, Iroha EO (2006). Heamatological abnormalities associated with paediatric HIV/AIDS in Lagos. Ann Trop Paediatr..

[14] Brian S Eley, Sive AA, Shuttleworth M, Hussey GD (2002). A prospective, cross-sectional study of anaemia and peripheral iron status in antiretroviral naive, HIV-1 infected children in Cape Town, South Africa. BMC Infect Dis..

[15] Swaminathan S, Padmapriyadarsini C, Sukumar B, Iliayas S, Kumar SR, Triveni C, Gomathy P, Thomas B, Mathew M, Narayanan PR (2008). Nutritional status of persons with HIV infection, persons with HIV infection and tuberculosis, and HIV-negative individuals from southern India. Clin Infect Dis..

[16] Otieno RO, Ouma C, Ong'echa JM, Keller CC, Waindi FR (2006). Increased severe anemia in HIV-1-exposed and HIV-1-positive infants and children during acute malaria. American National Library of Medecine National Institutes of Health, SIDA..

